# Population Genetic Analysis Infers Migration Pathways of *Phytophthora ramorum* in US Nurseries

**DOI:** 10.1371/journal.ppat.1000583

**Published:** 2009-09-18

**Authors:** Erica M. Goss, Meg Larsen, Gary A. Chastagner, Donald R. Givens, Niklaus J. Grünwald

**Affiliations:** 1 Horticultural Crops Research Laboratory, USDA ARS, Corvallis, Oregon, United States of America; 2 Washington State University Research and Extension Center, Puyallup, Washington, United States of America; 3 USDA APHIS PPQ, Fort Collins, Colorado, United States of America; University of Melbourne, Australia

## Abstract

Recently introduced, exotic plant pathogens may exhibit low genetic diversity and be limited to clonal reproduction. However, rapidly mutating molecular markers such as microsatellites can reveal genetic variation within these populations and be used to model putative migration patterns. *Phytophthora ramorum* is the exotic pathogen, discovered in the late 1990s, that is responsible for sudden oak death in California forests and ramorum blight of common ornamentals. The nursery trade has moved this pathogen from source populations on the West Coast to locations across the United States, thus risking introduction to other native forests. We examined the genetic diversity of *P. ramorum* in United States nurseries by microsatellite genotyping 279 isolates collected from 19 states between 2004 and 2007. Of the three known *P. ramorum* clonal lineages, the most common and genetically diverse lineage in the sample was NA1. Two eastward migration pathways were revealed in the clustering of NA1 isolates into two groups, one containing isolates from Connecticut, Oregon, and Washington and the other isolates from California and the remaining states. This finding is consistent with trace forward analyses conducted by the US Department of Agriculture's Animal and Plant Health Inspection Service. At the same time, genetic diversities in several states equaled those observed in California, Oregon, and Washington and two-thirds of multilocus genotypes exhibited limited geographic distributions, indicating that mutation was common during or subsequent to migration. Together, these data suggest that migration, rapid mutation, and genetic drift all play a role in structuring the genetic diversity of *P. ramorum* in US nurseries. This work demonstrates that fast-evolving genetic markers can be used to examine the evolutionary processes acting on recently introduced pathogens and to infer their putative migration patterns, thus showing promise for the application of forensics to plant pathogens.

## Author Summary

Sudden oak death, caused by the fungus-like pathogen *Phytophthora ramorum*, has caused devastating levels of mortality of live oak and tanoak trees in coastal California forests and in urban and suburban landscapes in the San Francisco Bay Area. This pathogen also causes non-lethal disease on popular ornamental plants, including rhododendrons, viburnums, and camellias. *P. ramorum* was discovered in California in the late 1990s and is exotic to the United States. Recently, presence of the disease in wholesale nurseries in California, Oregon, and Washington has led to shipments of diseased plants across the US, thus risking the introduction of the pathogen to other vulnerable forests. We examined the genetic diversity of this pathogen in US nurseries in order to better understand its evolution in nurseries and movement between states. We found that California populations were genetically different enough from Oregon and Washington populations that infestations of the pathogen found in nurseries in other states could be distinguished as having originated from California or the Northwest. Our inferences were consistent with trace forward investigations by regulatory agencies.

## Introduction

Plant pathogens that have been introduced to a new environment may be characterized by low genetic diversity due to a genetic bottleneck experienced during the process of introduction and establishment, given that only one or a few genotypes are usually introduced [1]–[5]. Genetic diversity may also be lower on the margins of an epidemic or in founder compared to older populations [6]–[9]. In some cases the absence of a mating type may limit the pathogen to clonal reproduction and contribute to its reduced genetic diversity, yet clonality does not necessarily prevent continued evolution. *Phytophthora infestans*, causal agent of potato and tomato late blight, is a well known example of a plant pathogen able to adapt while reproducing clonally, as observed by changing virulence on host cultivars [10]. Stepwise evolution of new pathotypes in a single clonal lineage has also been observed for stripe rust of wheat, *Puccinia striiformis* f.sp. *tritici*, in Australia and New Zealand [4]. The increasing development and availability of polymorphic neutral genetic markers have allowed for detailed exploration of the genetic variation contained within clonal lineages [11]–[13].

Genetic markers are also beginning to be used for forensic purposes in human pathogens. Microbial forensics is “the detection of reliably measured molecular variations between related microbial strains and their use to infer the origin, relationships, or transmission route of a particular isolate” [14]. This approach has been taken to examine high-profile HIV outbreaks and transmission events [15],[16] and characterize anthrax strains associated with bioterror attacks [17],[18]. Forensics requires a sound scientific foundation, including knowledge of the genetic diversity within and among populations of the organism of interest and the evolutionary forces and genetic mechanisms that shape this diversity [19],[20]. The population genetic base required for forensic work remains weak for many plant pathogens that pose economic or environmental threats [19].

*Phytophthora ramorum*, the causal agent of sudden oak death, was recently introduced to North America and is responsible for the rapid decline of forest populations of tanoak (*Lithocarpus densiflorus*) and coast live oak (*Quercus agrifolia*) in northern California coastal forests and parts of coastal southern Oregon [21],[22]. *P. ramorum* is also a foliar and twig pathogen on common ornamentals, such as *Rhododendron*, *Viburnum*, *Pieris*, and *Camellia*. Thus, *P. ramorum* has been found in nurseries in North America and Europe, and nursery shipments have been implicated in the movement of the pathogen. There is serious concern about the inadvertent transfer of *P. ramorum* to other susceptible ecosystems, such as the Appalachians [23]. *P. ramorum* has had significant economic and societal impacts [22],[24],[25].

*P. ramorum* is a diploid oomycete, located in the kingdom Stramenopila along with diatoms, golden-brown algae, and brown algae [26],[27]. Fast-evolving microsatellites in *P. ramorum* have confirmed the clonal reproduction of this pathogen and have proved valuable for examining its population structure [12],[13],[28],[29]. Three distinct clonal lineages of *P. ramorum* have been found in nurseries [28],[30]. These lineages appear to have been evolutionarily isolated for at least 100,000 years [31], which together with their initial geographic distributions suggests that there were three introductions of this pathogen to North America and Europe [32]. The lineages have been given the names NA1, NA2, and EU1 by consensus agreement within the *P. ramorum* research community [33]. The NA1 lineage has been the most frequently isolated lineage from US nurseries and is the cause of oak and tanoak mortality in US forests [13],[28]. The EU1 lineage was initially confined to European nurseries, but is now also found in European parks and North American nurseries [34]–[36]. The third lineage, NA2, has only been documented in North American nurseries [28],[36]. *P. ramorum* is self-sterile; sexual reproduction requires contact between two different mating types. All tested NA1 and NA2 isolates have been mating type A2 and EU1 isolates mating type A1 with the exception of rare finds of A2 in Belgium [37]. Sexual reproduction has not yet been observed in nurseries where both mating types have been found [34].

Most of the *P. ramorum*-positive nurseries have been in California, Oregon, and Washington, where annual inspection and sampling is required for nurseries that ship interstate and contain host or associated host plants on the *P. ramorum* host lists per the Federal Interim Rule of 2007 (7 CFR 301.92). West Coast nurseries that ship non-host nursery stock interstate are also required to be inspected annually. When found, infected plants are quarantined and destroyed under the authority of the Plant Protection Act of 2000. *P. ramorum* has also been found in states that received shipments from infected West Coast nurseries. For example, shipments of millions of potentially infected plants were made from a large California nursery to over 1,200 nurseries in 39 states in 2004 [25]. When a nursery has been confirmed as infested with *P. ramorum* and it has been determined that the nursery shipped potentially infected *P. ramorum* host or associated host plants, the nursery is required to provide to the US Department of Agriculture's Animal and Plant Health Inspection Service (USDA APHIS) a list of all host and associated host plants that were shipped from the nursery during the preceding 12 months. A trace forward protocol (http://www.aphis.usda.gov/plant_health/plant_pest_info/pram/) is implemented to determine whether the receiving nurseries or landscapes have become infested. Similarly, a trace back protocol is implemented at the infested shipping nursery to investigate the potential source of *P. ramorum*.

Previous studies examining neutral genetic variation in nursery populations of *P. ramorum* using mitochondrial DNA sequence, AFLP, or microsatellites have focused on the broad diversity of a worldwide sample of *P. ramorum* isolates [28],[30],[38] and specifically on Oregon [13], California [12], or West Coast [29] populations using isolates collected through 2005. These studies have shown genetic similarity between 2004 nursery isolates and early California forest infestations [12] and migration among West Coast populations in the first half of this decade [29]. The Oregon forest population is an apparent exception to the frequent migration between California, Oregon, and Washington, as it is genetically differentiated from both California forest and Oregon nursery populations [13]. Thus far, microsatellites have been the most informative markers for examining population structure and migration.

Here we report on the population genetic analysis of *P. ramorum* in US nurseries using 279 isolates collected from infected nurseries from across the US between 2004 and 2007. There is interest in the *P. ramorum* community in using genetic markers to link new detections of *P. ramorum* in both nursery and wildland settings to possible sources; therefore, we typed microsatellite loci known to show variation within and between the *P. ramorum* clonal lineages to examine their utility in confirming or contributing to trace forward and trace back investigations and, more generally, the potential for forensic analysis of *P. ramorum*. We specifically address four major questions regarding nursery populations of *P. ramorum*: 1) Do nursery populations show genetic diversity and population structure or are they dominated by a single dominant or founding genotype? 2) Are West Coast infestations more genetically diverse than those in other states, as might be expected if infestations are older and effectively larger in Oregon, Washington, and California? 3) Have the populations of the West Coast states changed between 2004 and 2007 in a way that would indicate that eradication measures have or have not been effective? 4) Can we use these genetic markers to infer the major migration pathways and potential sources of recent migrants?

## Results

### Genetic variation by state and year

**Figure 1:**
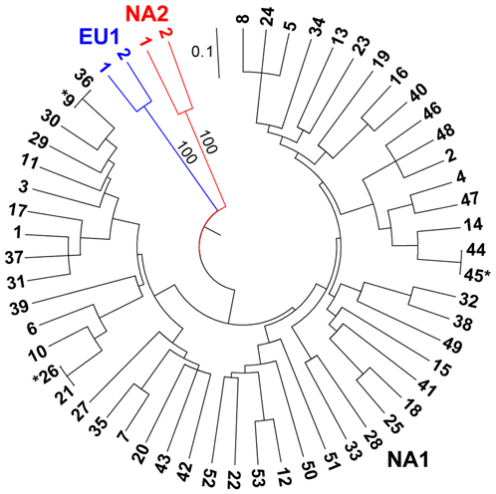
UPGMA dendrogram of genetic distance among all observed multilocus genotypes. Multilocus genotypes (MGs) are labeled as in Table S2 for NA1 isolates. Only two NA2 and EU1 multilocus genotypes were found. NA1 genotypes with null alleles are marked with asterisks. Support values greater than 70% using 1,000 bootstrap samples are shown.

All 279 isolates produced multilocus genotypes that could be unambiguously assigned to one of the three known *P. ramorum* lineages and no recombinant multilocus genotypes were observed that would be indicative of sexual reproduction between lineages. Thirty-four EU1 isolates and 17 NA2 isolates were identified in the sample (Table 1). EU1 isolates were found in California (CA), Oregon (OR), and Washington (WA) and produced two genotypes (Figure 1), which differed by two repeats at locus 64. OR and WA isolates were all identical, while all but one of the CA isolates were the second genotype. All of the NA2 isolates were from WA and produced identical genotypes except for one isolate from 2004 (Table 1, Figure 1), which differed by one repeat at both alleles of locus PrMS43a.

**Table 1:** Summary of the genetic diversity of *P. ramorum* nursery isolates by state and year collected.

Sample origin^a^	Year	NA1^b^						EU1		NA2		Source^c^
State		Isolates	MGs	G	E_5_	GR	AR	Isolates	MGs	Isolates	MGs	
Alabama (AL)	?	2	2	-	-	-	-					8
Arkansas (AR)	?	1	1	-	-	-	-					8
California (CA)	2004	4	3	-	-	-	-					1,9
	2006	12	7	4.80	0.80	3.85	1.66	3	1			
	2007	20	4	1.90	0.61	2.30	1.29	4	2			
Colorado (CO)	2004	1	1	-	-	-	-					8
Connecticut (CT)	2004	5	4	3.57	0.92	4.00	1.60					5
	2006	1	1	-	-	-	-					
Florida (FL)	2004	4	3	-	-	-	-					4,7,8
	2006	2	2	-	-	-	-					
	2007	4	2	-	-	-	-					
Georgia (GA)	2004	29	10	4.50	0.66	3.53	1.66					8
Louisiana (LA)	2004	1	1	-	-	-	-					8
Maryland (MD)	2004	1	1	-	-	-	-					8
Mississippi (MS)	2007	1	1	-	-	-	-					7
N. Carolina (NC)	2004	2	1	-	-	-	-					8
New Mexico (NM)	2004	1	1	-	-	-	-					8
Oregon (OR)	2004	5	3	2.27	0.80	3.00	1.80					6
	2005	7	5	4.45	0.93	4.05	1.81	12	1			
	2006	10	5	3.85	0.85	3.73	1.70	3	1			
	2007	5	5	5.00	1.00	5.00	1.80	8	1			
Pennsylvania (PA)	2004	1	1	-	-	-	-					3
S. Carolina (SC)	2004	2	2	-	-	-	-					8
Tennessee (TN)	2004	1	1	-	-	-	-					8
Texas (TX)	2004	9	7	5.40	0.84	4.29	1.72					8
Virginia (VA)	2004	5	4	3.57	0.92	4.00	1.80					8
Washington (WA)	2004	29	18	13.79	0.98	4.62	2.15			2	2	2,8
	2005	23	11	9.28	0.92	4.42	2.15			2	1	
	2006	15	10	6.08	0.73	4.34	2.00			1	1	
	2007	21	8	3.71	0.68	3.27	1.50	4	1	9	1	
	?	4	3	-	-	-	-			3	1	

a For each state and year the number of isolates sampled and the number of resulting multilocus genotypes (MGs) for each clonal lineage is given. For NA1 samples with 5 or more isolates, indices of diversity were calculated.

b Diversity indices for NA1 samples: G, Stoddart and Taylor's genotypic diversity index; E_5_, index of evenness; GR, genotypic richness expected for a sample size of five isolates; AR, allelic richness expected for a sample size of five isolates.

c Source of isolates: (1) C. Blomquist, California Department of Food and Agriculture; (2) G. Chastagner, Washington State University; (3) M. Garbelotto, University of California Berkley; (4) R. Leahy, Florida Department of Agriculture and Consumer Services; (5) R. Marra, Connecticut Agricultural Experiment Station; (6) N. Osterbauer, Oregon Department of Agriculture; (7) M. Palm, USDA APHIS; (8) J. Ristaino, North Carolina State University; (9) D. Rizzo, University of California Davis.

The NA1 lineage was the most common and genetically variable lineage in US nurseries, found in all sampled states. We found 53 different multilocus genotypes among the 228 NA1 isolates, including three genotypes with null alleles at PrMS43b (Figure 1, Tables S1 and S2). Unique to the NA1 lineage was apparent uniform homozygosity at loci PrMS39b, PrMS43a, and PrMS43b. These loci also exhibited high numbers of alleles among NA1 isolates relative to the other genotyped loci (Table S1). Loss of heterozygosity was observed for two isolates at locus PrMS45 and one isolate at locus 64.

Sample sizes from many states were very small, e.g. one isolate from one infested nursery in the state (Tables 1 and S3). For sample sizes up to about 15 isolates, there was a positive linear relationship between sample size and number of resulting multilocus genotypes, such that for the NA1 clonal lineage every five additional isolates produced around 3 additional multilocus genotypes (Figure S1). The relationship between sample size and genotypes changed at higher sample sizes and the number of multilocus genotypes was instead correlated with the number of infected nurseries in the state.

The lineages are separated by large genetic distances (Figure 1) and reproduction appears to be completely clonal [12],[13],[28], therefore the three lineages were considered separately. Furthermore, the paucity of EU1 and NA2 isolates and genotypes precluded the need for extensive analysis of these lineages and hence our analyses focused on NA1 isolates. We examined the genotypic diversity, genotypic evenness, and genotypic and allelic richness of NA1 samples by state and year for those with sample sizes of five or more isolates (Table 1). Importantly, given the variation in sample sizes among states, we used rarefaction to estimate genotypic and allelic richness for a standardized sample size of five isolates. Interestingly, genotypic richness in the Connecticut (CT), Georgia (GA), Texas (TX), and Virginia (VA) samples were at levels seen in the West Coast states. Evenness is expected to be influenced by differences in sampling intensity, but tended to decrease over the sampled years in CA and WA. Private alleles were found in OR, WA, TX, and VA. A larger number of states produced multilocus genotypes that were not observed elsewhere (Table S2).

**Figure 2:**
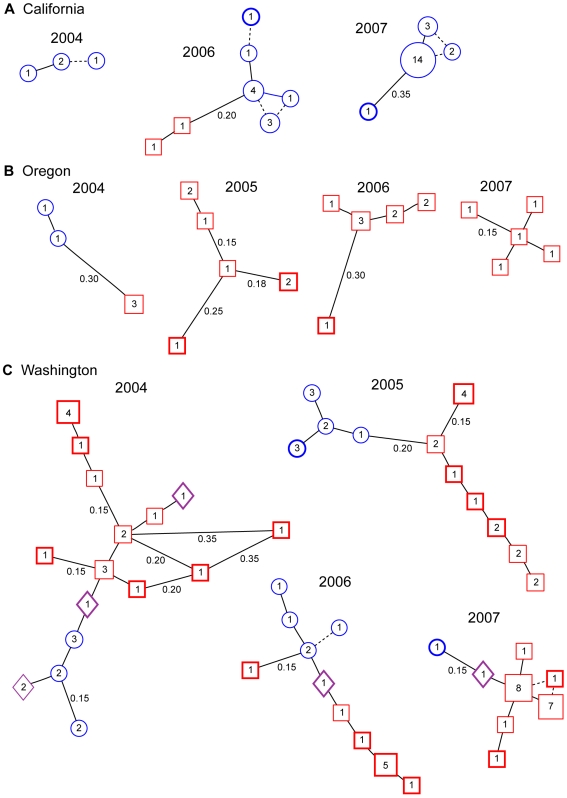
Minimum spanning networks for West Coast NA1 populations. A. California, B. Oregon, and C. Washington. For constructing the networks multilocus genotypes were collapsed to multilocus haplotypes, which are represented by circles, squares, or diamonds containing the number of associated isolates and sized in proportion to haplotype frequency. Blue circles and red squares represent the two different groups identified by Structure. Purple diamonds are haplotypes that could not be assigned to one group or the other with high confidence (>75% probability). Bolded haplotypes are those that were found in only that state (some haplotypes found only in Washington were seen in multiple years). Branches are proportional to Bruvo *et al.*'s [56] genetic distance and are labeled with distance if different than 0.10. Broken lines connect haplotypes that only differ by null alleles at one locus. Loops in the networks indicate multiple tied minimum spanning trees.

Minimum spanning networks revealed qualitative differences among states and years for the West Coast (Figure 2). By 2007, samples from all three states produced relatively compact networks, indicating that these populations had been limited to a small number of mostly closely related genotypes. The change over time was most evident in the Washington networks, in which there were long chains of genotypes prior to 2007. Private genotypes were generally on the margins of the networks and sometimes were only distantly related to the other genotypes, suggesting that they were either the result of rare mutation events or were immigrants from locations with intermediate genotypes.

**Figure 3:**
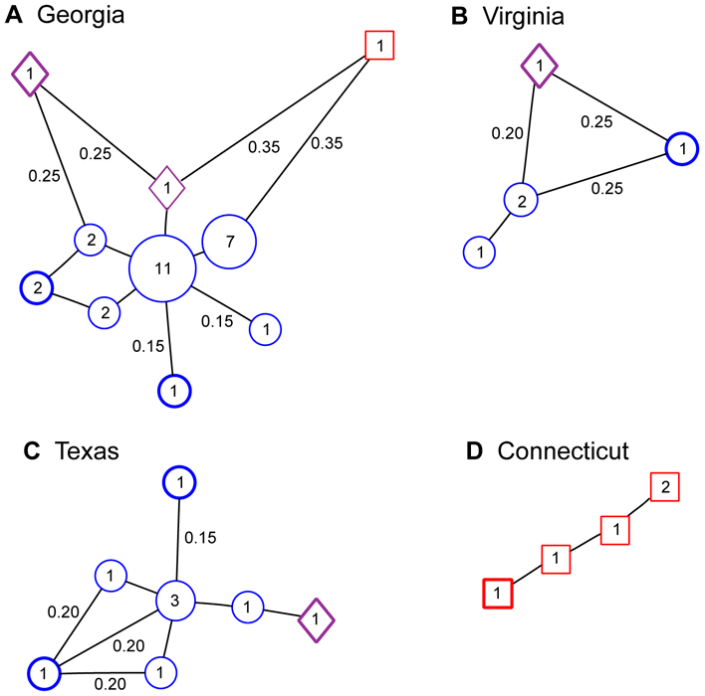
Minimum spanning networks for four 2004 NA1 populations. A. Georgia, B. Virginia, C. Texas, and D. Connecticut. Multilocus genotypes were collapsed to multilocus haplotypes, which are represented by circles, squares, or diamonds containing the number of associated isolates and sized in proportion to haplotype frequency. Blue circles and red squares represent the two different groups identified by Structure. Purple diamonds are haplotypes that could not be assigned to one group or the other with high confidence (>75% probability). Bolded haplotypes are those that were found in only that state. Branches are proportional to Bruvo *et al.*'s [56] genetic distance and are labeled with distance if different than 0.10. Loops in the networks indicate multiple tied minimum spanning trees. For GA, TX, and VA the most common haplotype corresponds to MG 1 in Table S2. The most common haplotype for CT is MG 2.

We also examined the minimum spanning networks for other states represented by five or more isolates to compare them to the West Coast states (Figure 3). These networks generally showed populations of closely related genotypes. The most common multilocus haplotype in each of the four networks corresponded to one of the two most common multilocus genotypes in the overall sample (either MG 1 or 2 in Table S2) and may thus be the founding genotype. The outlying haplotypes in the networks were often private genotypes.

We tested for significant genetic variation among West Coast states and years using analysis of molecular variance. We found significant variation among years within states, but more variation among states and within states and years (Table 2). Examination of CA, OR, and WA individually showed that variation among years accounted for 0% (*P* = 0.27), 3.0% (*P* = 0.13), and 4.9% (*P*<0.0001) of the total variation, respectively. When data were clone corrected there was significant variation among states but not among years within states (Table 2).

**Table 2:** Analysis of molecular variance (AMOVA) among California, Oregon, and Washington samples.

Source of variation	d.f.	Sum of squares	Percentage of variation	*P*	Fixation indices
Among states	2 (2)	55.5 (14.7)	16.2 (7.3)	<0.001 (0.003)	0.16 (0.07)
Among years within states	8 (8)	23.2 (12.6)	3.1 (−0.3)	<0.001 (ns)	0.04 (−0.003)
Within states and years	289 (147)	418.7 (242.1)	80.7 (93.0)	<0.001 (0.015)	0.19 (0.07)

Clone corrected values are shown in parentheses.

### NA1 population structure and migration

**Figure 4:**
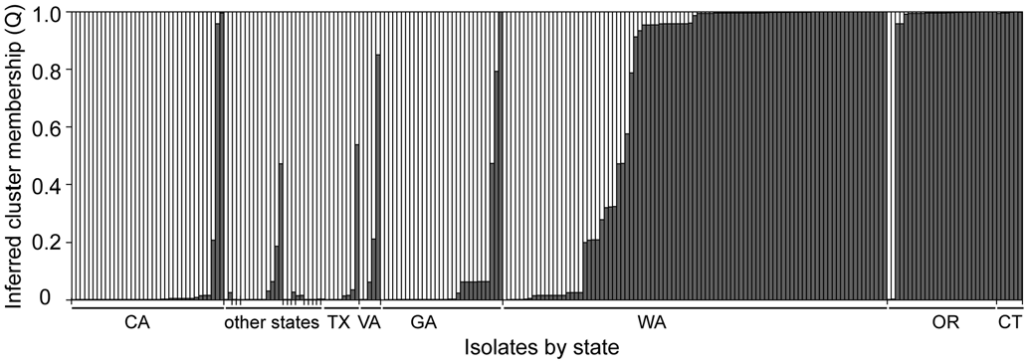
Clustering of isolates by Structure 2.2 into two underlying groups. For each state isolates are ordered by their probability of membership in the group common among Connecticut, Oregon, and Washington isolates. The order of the “other states”, from left to right, is AL, AR, CO, FL, LA, MD, NC, NM, PA, SC, and TN. See Table 1 for state abbreviations.

**Figure 5:**
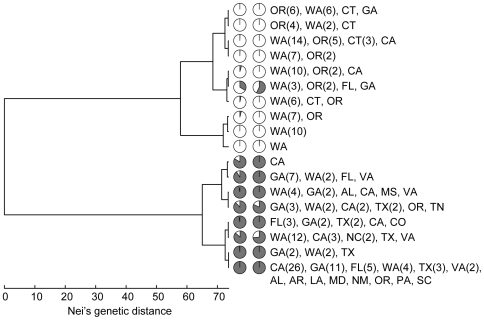
Comparison of the genetic groups identified by three clustering algorithms. The UPGMA dendrogram was produced for the 18 clusters identified by BAPS 5.2. The membership of each cluster is indicated to the far right by state, with the number of isolates if greater than one in parentheses. Pie charts show the group assignment of the isolates in each BAPS cluster by Structure 2.2 (left) and k-means clustering (right). See Table 1 for state abbreviations.

**Figure 6:**
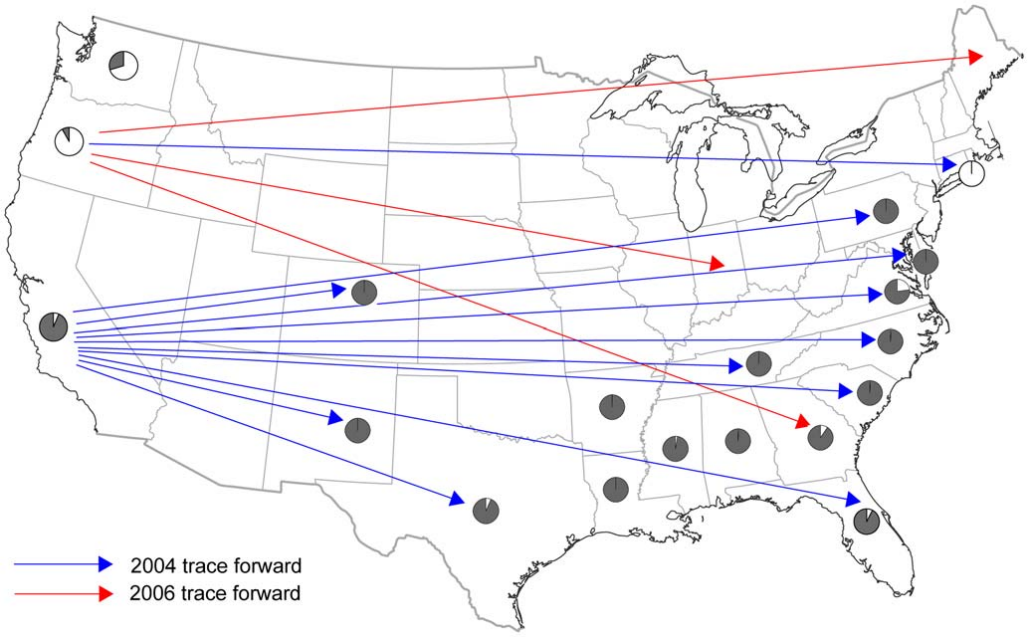
NA1 migration pathways. Pie charts show the distribution of the two groups of NA1 isolates, as identified by Structure, among sampled states. Arrows indicate confirmed *P. ramorum*-positive nursery trace forwards. Blue arrows are 2004 trace forwards and red arrows are 2006 trace forwards. There were no confirmed trace forwards in 2005 or 2007.

Structure 2.2 and BAPS 5.2 were used to cluster NA1 isolates, without regard to state or year of isolation, into underlying groups. The Structure analysis produced the highest likelihood for two groups (posterior probability that K = 2 was 1.00). AMOVA confirmed significant variation between these groups, which accounted for 33% of the variation. Ten isolates could not be assigned to one or the other group with a probability greater than 0.75 and 31 isolates were not assigned with a probability greater than 0.95 (Figure 4). The optimal partitioning of isolates by BAPS produced 18 clusters (Figure 5). However, these 18 clusters formed two overall groups that largely coincided with the two Structure groups (Figure 5). AMOVA on the BAPS groups indicated that the two overall groups were responsible for 27% of the variation and the clusters within the larger groups explained another 27% of the variation. K-means clustering of individuals based on either allele frequency or AMOVA also produced the best result for two groups based on Calinski and Harabasz's pseudo-F [39]. Differences in group assignment between Structure, BAPS, and k-means clustering were limited to twelve isolates, all of which produced low posterior probabilities for group assignment in Structure. Many states were represented by mostly one group or the other, but there were also mixed populations (Figures 4 and 6). Both groups were represented in WA in all years, OR in 2004, CA in 2006, and GA with high probability. Structure outputs the overall allele frequencies and frequencies within each resulting group, which showed that particular loci and alleles were highly influential in determining group assignments (Table 3). For example, allele 246 of locus PrMS39b had an overall frequency of 0.303, but a frequency of 0.936 in group 2.The influential alleles differed by only one repeat from each other, suggesting that these groups may not be robust to repeated and reverse mutation.

**Table 3:** Influential loci and alleles in Structure 2.2 analysis.

Locus	Allele^a^	Allele frequency	Group 1 frequency	Group 2 frequency
PrMS39b	246	0.303	0.046	0.936
	250	0.443	0.835	0.039
PrMS43a	372	0.206	0.089	0.728
	368	0.246	0.492	0.112
PrMS43b	489	0.251	0.218	0.658
	485	0.288	0.662	0.221

a Only alleles with large representations in one or the other group are shown.

**Figure 7:**
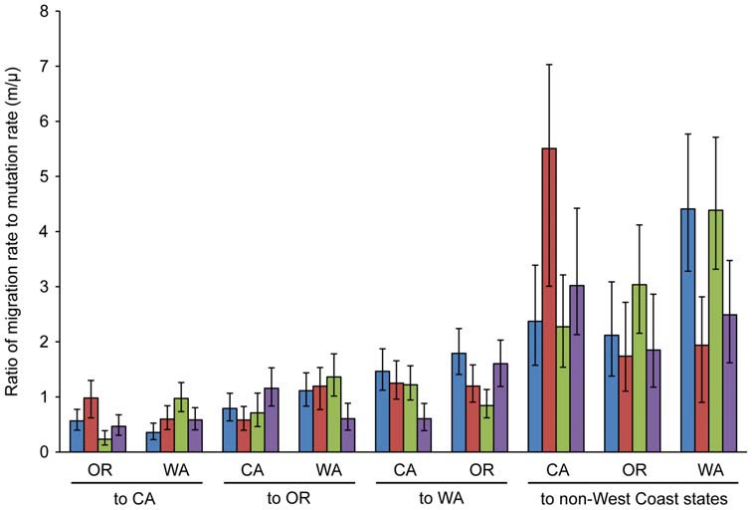
Estimates of relative rates of immigration to mutation (m/µ). The ratio of immigration rate to mutation rate was estimated for each of four populations. Isolates were combined across years for CA, OR, and WA, and combined across both years and states for a population of all non-West Coast isolates. The migration model allowed the three West Coast populations to both send and receive migrants, but the fourth population to only receive immigrants. Bars show the maximum likelihood estimate of the parameter for four independent runs of the program Migrate (indicated by different colors). Error bars indicate 95% confidence intervals.

The relative rates of immigration to mutation among West Coast states and from these states eastward were estimated using a coalescent-based approach, as implemented in the program Migrate. We used a migration model in which the three West Coast source populations could both send and receive migrants, but the combined population representing all other states could only receive immigrants. This migration model is consistent with nursery industry shipment patterns. The ratio of immigration rate to mutation rate (m/µ) tended to be higher for the non-West Coast sample, but with a large amount of uncertainty in the estimates (Figure 7). Many of the estimates were not significantly greater than 1.0, indicating that mutation and drift were often more important than migration in generating population genetic variation.

### Trace forwards

When a nursery that ships *P. ramorum* host and associated host plants out of state is confirmed to be infested with *P. ramorum*, the USDA APHIS trace forward protocol is implemented by the receiving state(s). Shipping records are obtained for all host and associated host plants that were shipped in the preceding 12 months. These shipping records are used to conduct inspections to determine whether receiving nurseries or landscapes have become infested. Trace forward shipments from *P. ramorum* infested nurseries in CA, OR and WA to non-West Coast states resulted in the detection of *P. ramorum* in 12 states from 2004 to 2007 (Figure 6). In 2004, all but three of the confirmed trace forward detections originated in CA. The remaining three were from OR to CT (2 detections) and MD (1 detection). Additional states received shipments from *P. ramorum* infested nurseries; however, the movement of any infected plants was not determined or confirmed.

## Discussion

Our analysis of the genotypic diversity of *P. ramorum* isolates from US nurseries revealed two genetic groups in the NA1 lineage. The composition of these groups suggests that many of the isolates collected in non-West Coast states were associated with California genotypes whereas the Connecticut infestation more closely resembled Oregon and Washington genotypes. This is in agreement with trace forward analyses by USDA APHIS, which established major shipments of *P. ramorum*-positive plants from California to nurseries across the country and smaller shipments from an Oregon nursery to Connecticut in 2004. The 2004 California shipments also sent *P. ramorum*-positive plants to Oregon and Washington, perhaps explaining representation from both genetic groups in these states' samples. Migration between all three West Coast states was also inferred by Prospero *et al.* [29] based on genotyping of California forest isolates and Oregon and Washington nursery isolates collected from 2003 to 2005. Yet, the clustering of isolates into two groups appeared to be highly influenced by three loci that show rapid evolution in NA1. This suggests that over time isolates could mutate between groups and thus grouping based on these markers may not be robust in the long term. The states with representatives from both groups tended to be those with higher numbers of multilocus genotypes and higher genotypic diversities, which could be explained by either more migration to these states or larger populations with more opportunities for mutation. For example, the networks of Washington isolates included chains of genotypes differing by single mutational steps yet assigned to different groups, suggesting that these mixed populations could be the result of large and diverse infestations.

*P. ramorum* isolates from nineteen states were examined and only five states were found that did not contain the most common genotype in the overall sample (NA1 multilocus genotype 1). Two of these, Connecticut and North Carolina, produced isolates with the second and third most common NA1 genotype, respectively. This suggests that only a few genotypes may be responsible for initiating *P. ramorum* infestations across the US. This is again consistent with USDA APHIS analysis, which indicated that shipments of infected plant material occurred only a few times. *P. ramorum* is also present in nurseries in British Columbia, Canada [36],[40] and there has been movement of the pathogen between BC and West Coast states every year since 2003 based on USDA APHIS trace data.

The most genetically variable populations were on the West Coast, as expected based on the large number of infected nurseries that have been found in these states (Table S3), yet we also found relatively diverse samples when we had five or more isolates from other states. The observed variation is likely related to the number of infested nurseries sampled and perhaps also to how long the infestations went undiagnosed, information that we do not have. Georgia and Texas had 14 and 11 confirmed positive nurseries, respectively, which could help explain the observed levels of variation, but Connecticut had only three and Virginia two positive nurseries (Table S3). More extensive sampling within nurseries would be required to elucidate the population structure in infested nurseries as our results suggest that we did not achieve saturation in sampling the diversity of nursery populations. In general, rapid detection and eradication should result in small effective population sizes and low genetic diversities. As the genotyping of nursery isolates becomes increasingly routine, more samples per nursery are being retained for genotyping. In fact, sampling appeared to be nearing saturation in 2007 for California and Washington nurseries.

Providing an interesting contrast to the single genotype shared among many states, we identified 36 NA1 multilocus genotypes that were unique to a state. Destruction of infected plants should ensure that populations in individual nurseries do not have the opportunity to grow large and small populations are subject to genetic drift. The observed genetic diversity and number of private genotypes suggests that there is also rapid mutation following the founding of a new nursery population and little to no gene flow following initial introduction. Interestingly, in California, several recently established *P. ramorum* forest populations (<5 yrs old) were observed to be as diverse as older forest populations (>10 yrs) and the genetic distance among new populations was greater than that observed among older populations [12], suggesting that a similar process of rapid mutation, genetic drift, and limited gene flow may characterize newly founded populations in both forest and nursery environments.

From 2004 to 2007 NA1 populations in West Coast nurseries appeared to become increasingly dominated by a few closely related genotypes and in 2007 all three states produced compact minimum spanning networks. This pattern is particularly striking for Washington, from which we had the largest numbers of isolates and observed high genotypic and allelic richnesses, and suggests that in 2007 there were fewer nodes of infection or earlier detection and eradication of infections. In fact, West Coast states had many fewer *P. ramorum*-positive nurseries in 2007 than in previous years (Table S3).

Prospero *et al.* [13] examined *P. ramorum* isolates from Oregon nurseries collected in 2003 and 2004, finding four NA1 genotypes in 2003 and six in 2004. Although each year was dominated by two closely related genotypes, there were no genotypes in common between years, which suggested that the 2003 nursery infestations were eradicated and the 2004 infestations were new introductions. In our sample of Oregon nursery NA1 isolates from 2004 through 2007, we did not find significant genetic variation across years. Of 13 multilocus genotypes found in Oregon, 4 of these were found in more than one year and 3 additional genotypes differed by one repeat from a genotype found in multiple years. Thus, some genotypes may have persisted in Oregon nurseries. However, the most common Oregon multilocus genotype (NA1 MG 2) was found in 2004, 2005, and 2006 but not 2007.

California nursery populations were dominated by a single genotype (NA1 MG 1), comprising 20 of the 36 isolates from the state. Mascheretti *et al.* [12] found the same dominant genotype in their nursery sample, which was also a common genotype in the California forests. This genotype has been observed in nurseries since 2004, thus it is either not being eradicated from nurseries or is re-colonizing nurseries from forest populations.

Given the levels of heterozygosity observed at most of the microsatellite loci [13],[28] and in the nuclear genome [41], the consistent homozygosity at loci PrMS39b, PrMS43a, and PrMS43b is unexpected. Loci PrMS45 and 64 were also heterozygous in all but three isolates and had large differences in allele sizes, therefore this limited homozygosity was likely a result of mitotic recombination. Mitotic recombination generally refers to crossing-over during mitosis, which results in the loss of heterozygosity at all loci distal to the chromosomal breakpoint. Loss of heterozygosity may also be the result of mitotic gene conversion, in which case only a small segment of the chromosome is altered. Mitotic recombination is thought to be responsible for frequent observations of loss of heterozygosity in *P. infestans* allozymes [42] and *P. cinnamomi* microsatellites [11]. Mitotic gene conversion has been observed in *P. sojae* [43]. Mitotic recombination or gene conversion may also provide an explanation for the homozygosity at PrMS39b, PrMS43a, and PrMS43b, where it must occur at a very rapid rate as these are also fast-evolving loci. It is also possible that these three loci are hemizygous or heterozygous for a null allele [11] or that intermediate genotypes have simply not been sampled.

Mitotic recombination may purge deleterious mutations from *Phytophthora* populations in the absence of sexual reproduction and unmask recessive traits or advantageous new mutations [11],[42]. However, the eradication of infections in US nurseries results in small effective population sizes and populations likely to be structured by genetic drift rather than natural selection. The major effect of mitotic recombination on nursery populations may be to increase the genetic distance between isolates as new mutations are made homozygous and passed on to asexual progeny. The genetic diversity among populations that could conceivably be created by this process may benefit the pathogen in the long term if in the future these populations are allowed to grow unchecked, which would allow natural selection to weed out the more fit recombinants from the less fit. Meiotic recombination through sexual reproduction would further benefit these populations by breaking linkages between beneficial and detrimental mutations. Limiting the distribution of the EU1 lineage, which is primarily the A1 mating type, and its proximity to NA1 and NA2 lineages (A2 mating type) will reduce the possibility of sexual reproduction.

The EU1 clonal lineage has now been found in all three west coast states [34], yet detectable genetic diversity in both this lineage and the NA2 lineage remain low. This could be due to the hypervariability of several of the microsatellite loci in NA1 but not EU1 and NA2, the more recent introduction of EU1 and NA2 to North America, and/or smaller population sizes of these lineages in US nurseries compared to the NA1 lineage. The recent finding of a single nucleotide polymorphism in the mitochondrial DNA of the NA1 lineage implies that this lineage may have a larger effective population size than the other two lineages [30].

The rapid mutation rates of these microsatellite loci has proven valuable for population genetic analyses, but poses a challenge for forensic tracing of *P. ramorum* when mutation rates are as high as appears to be the case for the PrMS43a and PrMS43b loci in the NA1 lineage. For example, an isolate of interest may differ from a suspected source population at one of these loci, thus raising doubts about their connection. Alternatively, convergence through repeat or reverse mutations may also have caused some Washington isolates to cluster with isolates from California and other states, which could falsely imply a direct connection between states where there is none. On the other hand, the relative homogeneity of the EU1 and NA2 lineages in US nurseries may hinder genetic-based tracing of isolates in these lineages. Nevertheless, our results were consistent with trace forward analyses and thus these microsatellites should be informative when used in conjunction with other data. The identification of more microsatellite loci that exhibit variation within the clonal lineages would strengthen these inferences [32],[44],[45].

Continued genotyping of *P. ramorum* from nurseries will be necessary to track the movement and diversification of the lineages and to identify new dominant genotypes, newly introduced lineages, or recombinant genotypes. As part of our efforts, the clonal lineage of each *P. ramorum* isolate genotyped, with permission from the provider of the isolate, is posted to a public website along with its county and state of origin at http://oregonstate.edu/~grunwaln/index.htm. Ongoing genotyping will also be valuable in evaluating how effective eradication efforts are in restricting migration, lowering effective population size, and increasing the effect of genetic drift.

## Materials and Methods

### Isolates

Isolates of *P. ramorum* were obtained from scientists with State Departments of Agriculture, the US Department of Agriculture's Animal and Plant Health Inspection Service, universities and research institutions as new or recurring findings of infected nurseries occurred. Newly infected sites are subject to federal quarantine and could not be systematically sampled. Thus sampling intensity likely varied by state. For example, isolates from non-West Coast states may each represent one infested nursery, whereas recent samples from OR and WA include multiple isolates per nursery. Isolates for which we had detailed host information came from *Camellia japonica*, *C. sasanqua*, *C. bonsai*, *Kalmia latifolia*, *Laurus noblis*, *Osmanthus heterophyllus*, *O. fragrans*, *Pieris japonica*, *Rhododendron* spp., *Viburnum tinus*, and from soil and water baits. The 2004 shipments from CA to 39 states contained *Camellia* species. We do not know how many nurseries with recurrent infestations that were sampled over 2 or more years are represented in our dataset.

Upon receipt, isolates were transferred to cleared 20% V8 agar medium (200 ml V8 juice; 2 g CaCO_3_; 30 mg/L β-sitosterol (EMD Chemicals, Inc., San Diego, CA); 15 g agar; 800 ml deionized water) and stored at 20°C in the dark. All isolates were handled following the standard operating procedures associated with corresponding USDA APHIS permits and an exemption from the Director of the Oregon Department of Agriculture for work with *P. ramorum* under containment conditions.

### Microsatellite genotyping

Six microsatellite loci were genotyped that had previously shown variation among isolates within the *P. ramorum* clonal lineages: PrMS39b, PrMS43a, PrMS43b, PrMS45 [13], 18, and 64 [28]. These loci are also differentiated between lineages. Three additional loci that are invariable within lineages, PrMS6, Pr9C3, and PrMS39a, were also genotyped. Genomic DNA was extracted from mycelia grown in cleared 20% V8 broth using the FastDNA SPIN kit (MP Biomedicals LLC, Solon, OH) following the protocol for yeast, algae, and fungi. Loci were amplified using primers and protocols as outlined in [28] and [13]. PrMS6, Pr9C3, PrMS39a and b, and PrMS45 were amplified using a PCR program of 1 cycle of 92°C for 2 min, followed by 30 cycles of 92°C for 30 s, 52°C for 30 s, 65°C for 30 s, and 1 cycle of 65°C for 5 min. Fluorescent multiplex PCR reactions were performed in 10-µL volumes with the following final concentrations: 1× GenScript PCR Buffer (10 mM Tris-HCl; 50 mM KCl; 1.5 mM MgCl_2_; 0.1% Triton X-100 buffer), 0.2 µM dNTPs, 3–6 µM of primer pairs, 0.5 U GenScript Taq DNA polymerase (Genscript Corporation, Piscataway, NJ), and 0.5 µL (∼50 ng) DNA template. Loci PrMS43a and b were amplified using the following PCR program: 1 cycle of 92°C for 2 min, 35 cycles of 92°C for 30 s, 52°C for 30 s, and 72°C for 1 min, and 1 cycle of 72°C for 45 min. The final concentrations of the reaction mixture for PrMS43 (10 µL volume) were 1× PCR Buffer, 0.4 µM dNTPs, 0.3 µM forward and reverse primers, 1.0 U DNA polymerase, and 0.5 µL DNA template. Loci 18 and 64 were amplified with the PCR program: 1 cycle of 94°C for 2 min, 30 cycles of 94°C for 20 s, 55°C for 20 s, and 72°C for 30 s min, and 1 cycle of 72°C for 10 min. The final concentrations of the 10 µL reaction mixture for 18 and 64 were 1× PCR Buffer, 0.2 µM dNTPs, 0.2 µM forward and reverse primers, 0.5 U DNA polymerase, and 0.5 µL DNA template.

Three isolates were used as positive controls in identification of the three clonal lineages and to ensure consistency among runs: PR-04-001 (aka 2027.1, lineage NA1 from Curry County, Oregon), PR-04-020 (aka 03-74-D12-A, EU1 from an Oregon nursery), and PR-04-015 (aka wsda3765, NA2 from a Washington nursery). PCR products were sized using capillary electrophoresis on an 3100 Avant Genetic Analyzer (Applied Biosystems, Foster City, CA) using the internal size-standard LIZ 500 (Applied Biosystems). Results were analyzed using GeneMapper 3.7 software packages (Applied Biosystems). Genotyping was replicated for a subset of isolates with independent DNA extractions, PCR, and sizing of fragments. Reproducibility of novel allele sizes was confirmed.

### Analysis

Genetic distances among all identified multilocus genotypes were calculated over eight of the nine loci using Wright's modification of Roger's genetic distance [46],[47] as implemented in the program TFPGA [48]. PrMS39a was excluded from the calculation because it was invariable in NA1 isolates and did not amplify in the other two lineages. Null alleles were coded as missing data. A UPGMA dendrogram was inferred from the distance matrix and visualized using MEGA version 4 [49]. Bootstrapping of data was conducted in TFPGA using 1,000 permutations.

For the NA1 lineage and states and years with sample sizes of at least five isolates, we estimated multilocus genotypic diversity using Stoddart and Taylor's index *G* [50], multilocus genotypic evenness (the distribution of genotypes in a sample) using the index *E*_5_ [51],[52], and multilocus genotypic richness and allelic richness (average number of alleles per locus) corrected for sample size using rarefaction as implemented in ADZE [53].

Analysis of molecular variance (AMOVA) [54] was conducted using Arlequin 3.1 [55] to test for significant variation among years in CA, OR, and WA. The analyses used the standard data setting and 10,000 permutations.

In order to examine genetic distances among isolates as measured by mutational differences, rather than mutation plus mitotic recombination, we collapsed the data to the haploid state. Three loci, PrMS39b, PrMS43a, and PrMS43b, were consistently homozygous. Loci 18 and 64 had two distinct size classes of alleles and only the larger of the two was variable among isolates for both loci. Thus when collapsed to haploid, the larger allele was retained. Three additional isolates appeared to exhibit mitotic recombination rather than mutation at otherwise uniformly heterozygous loci. These were two WA 2004 isolates (locus PrMS45) and a SC 2004 isolate (locus 64), multilocus genotypes 49, 53, and 35, respectively (Tables S1 and S2). These isolates were excluded from the haploid data set. PrMS45 was monomorphic across remaining NA1 isolates and PrMS6 and Pr9C3c were invariable within NA1.

To examine the relationships among isolates, minimum spanning networks were constructed using the genetic distance of Bruvo *et al.* [56], which incorporates microsatellite repeat number. Here, a distance of 0.10 is equivalent to one mutational step (one repeat) but larger distances do not strictly correspond to a given number of mutational steps. Genetic distance matrices were calculated for the three West Coast states for all available years and for the 2004 samples from CT, GA, TX, and VA using the haploid dataset. MINSPNET [57] was used to generate minimum spanning networks from genetic distance matrices. All tied trees were included in the network, which was visualized using the neato program in the Graphviz package [58].

To examine genetic structure in the NA1 sample, the clustering programs Structure 2.2 [59],[60] and BAPS 5.2 [61] were run using the haploid data set. For Structure 2.2 we used the no admixture model, because the NA1 lineage appears to be completely clonal, and assumed that allele frequencies among populations were correlated. However, very similar results were obtained using the admixture model and independent allele frequencies. Lambda was set to 1.0 and 100,000 MCMC replicates were used after a burn-in of 20,000. The number of underlying groups (K) was varied from 1 to 5 and replicated five times. The posterior probability of the most likely K was calculated assuming a uniform prior as described in the Structure 2.2 documentation. Genetic mixture analysis was run at the individual level in BAPS 5.2 for maximum number of populations (K) from 2 to 31, replicated 3 times. A UPGMA dendrogram of the resulting clusters was produced using Nei's distance as implemented by the program. AMOVA was conducted on the resulting Structure and BAPS clusters. Structure and BAPS results were also compared to those obtained from k-means clustering of individuals as implemented in Genodive [62], which does not assume Hardy-Weinberg equilibrium within populations.

Maximum likelihood estimates of the ratio of immigration rate to mutation rate (m/µ) for West Coast states compared to the non-West Coast sample were obtained using the program Migrate version 2.4.3 [63]–[65]. Isolates from all years were divided into four populations: CA, OR, WA, and all other states. All years of collection were combined to obtain larger population sizes for parameter estimates. The data were coded such that the homozygous loci had one missing allele, to account for the possibility of homozygosity by mitotic recombination rather than mutation. The analysis used a migration model in which the three West Coast source populations could both send and receive migrants, but the fourth combined population could only receive immigrants. We used the Brownian motion approximation to the stepwise mutation model and a search strategy of 10 short chains of 500 steps followed by 3 long chains of 10,000 steps at the default sampling increments with 3 heated chains using the adaptive heating scheme. The search strategy was replicated five times for each locus within each run such that the last chains of each replicate were combined for parameter estimation. Runs for which the profile likelihood calculation failed were discarded. A total of four runs were examined to account for possible variation among runs.

## Supporting Information

Table S1 Allele sizes at eight loci for each multilocus genotype (MG) observed within clonal lineages EU1, NA1, and NA2.
(0.07 MB PDF)

Table S2 Distribution of multilocus genotypes belonging to the NA1 clonal lineage.
(0.06 MB PDF)

Table S3 Confirmed *P. ramorum*-positive nursery-related sites (nurseries and residential landscapes, except for CA which is nurseries only) by state and year (http://www.suddenoakdeath.org).
(0.06 MB PDF)

Figure S1 Relationship between sample size and number of multilocus genotypes in the sample. Samples are isolates from one state in one year. The upper graph shows this relationship when multilocus genotypes from all three clonal lineages are considered. The lower graph shows the number of multilocus genotypes in the NA1 lineage only. Sample year is shown for the largest sample sizes to the right of the marker.
(0.29 MB TIF)

## References

[1] Bentley S, Pegg KG, Moore NY, Davis RD, Buddenhagen IW (1998). Genetic variation among vegetative compatibility groups of *Fusarium oxysporum* f. sp. *cubense* analyzed by DNA fingerprinting.. Phytopathology.

[2] Goodwin SB, Cohen BA, Fry WE (1994). Panglobal distribution of a single clonal lineage of the Irish potato famine fungus.. Proc Natl Acad Sci USA.

[3] Linde C, Drenth A, Wingfield MJ (1999). Gene and genotypic diversity of *Phytophthora cinnamomi* in South Africa and Australia revealed by DNA polymorphisms.. Eur J Plant Pathol.

[4] Steele KA, Humphreys E, Wellings CR, Dickinson MJ (2001). Support for a stepwise mutation model for pathogen evolution in Australasian *Puccinia striiformis* f.sp. *tritici* by use of molecular markers.. Plant Pathol.

[5] van Heerden SW, Wingfield MJ (2001). Genetic diversity of *Cryphonectria cubensis* isolates in South Africa.. Mycol Res.

[6] Mahoney EM, Milgroom MG, Sinclair WA, Houston DR (1999). Origin, genetic diversity, and population structure of *Nectria coccinea* var. *faginata* in North America.. Mycologia.

[7] Liu Y-C, Cortesi P, Double ML, MacDonald WL, Milgroom MG (1996). Diversity and multilocus genetic structure in populations of *Cryphonectria parasitica*.. Phytopathology.

[8] Brasier CM (1988). Rapid changes in genetic structure of epidemic populations of *Ophiostoma ulmi*.. Nature.

[9] Zhang N, Blackwell M (2002). Population structure of dogwood anthracnose fungus.. Phytopathology.

[10] Goodwin SB, Sujkowski LS, Fry WE (1995). Rapid evolution of pathogenicity within clonal lineages of the potato late blight disease fungus.. Phytopathology.

[11] Dobrowolski MP, Tommerup IC, Shearer BL, O'Brien PA (2003). Three clonal lineages of *Phytophthora cinnamomi* in Australia revealed by microsatellites.. Phytopathology.

[12] Mascheretti S, Croucher PJP, Vettraino A, Prospero S, Garbelotto M (2008). Reconstruction of the Sudden Oak Death epidemic in California through microsatellite analysis of the pathogen *Phytophthora ramorum*.. Mol Ecol.

[13] Prospero S, Hansen EM, Grünwald NJ, Winton LM (2007). Population dynamics of the sudden oak death pathogen *Phytophthora ramorum* in Oregon from 2001 to 2004.. Mol Ecol.

[14] Cummings CA, Relman DA (2002). Microbial forensics – “cross-examing pathogens”.. Science.

[15] de Oliveira T, Pybus OG, Rambaut A, Salemi M, Cassol S (2006). Molecular epidemiology: HIV-1 and HCV sequences from Libyan outbreak.. Nature.

[16] Metzker ML, Mindell DP, Liu XM, Ptak RG, Gibbs RA (2002). Molecular evidence of HIV-1 transmission in a criminal case.. Proc Natl Acad Sci USA.

[17] Keim P, Smith KL, Keys C, Takahashi H, Kurata T (2001). Molecular investigation of the Aum Shinrikyo anthrax release in Kameido, Japan.. J Clin Microbiol.

[18] Read TD, Salzberg SL, Pop M, Shumway M, Umayam L (2002). Comparative genome sequencing for discovery of novel polymorphisms in *Bacillus anthracis*.. Science.

[19] Fletcher J, Bender C, Budowle B, Cobb WT, Gold SE (2006). Plant pathogen forensics: capabilities, needs, and recommendations.. Microbiol Mol Biol Rev.

[20] Budowle B, Schutzer SE, Ascher MS, Atlas RM, Burans JP (2005). Toward a system of microbial forensics: from sample collection to interpretation of evidence.. Appl Environ Microbiol.

[21] Rizzo DM, Garbelotto M, Davidson JM, Slaughter GW, Koike ST (2002). *Phytophthora ramorum* as the cause of extensive mortality of *Quercus* spp. and *Lithocarpus densiflorus* in California.. Plant Dis.

[22] Rizzo DM, Garbelotto M, Hansen EM (2005). *Phytophthora ramorum*: integrative research and management of an emerging pathogen in California and Oregon forests.. Annu Rev Phytopathol.

[23] Venette RC, Cohen SD (2006). Potential climatic suitability for establishment of *Phytophthora ramorum* within the contiguous United States.. For Ecol Manage.

[24] Rizzo DM, Garbelotto M (2003). Sudden oak death: endangering California and Oregon forest ecosystems.. Front Ecol Environ.

[25] Frankel SJ (2008). Sudden oak death and *Phytophthora ramorum* in the USA: a management challenge.. Australas Plant Path.

[26] Baldauf SL (2003). The deep roots of Eukaryotes.. Science.

[27] Förster H, Coffey MD, Elwood H, Sogin ML (1990). Sequence analysis of the small subunit ribosomal RNAs of three zoosporic fungi and implications for fungal evolution.. Mycologia.

[28] Ivors K, Garbelotto M, Vries ID, Ruyter-Spira C, Te Hekkert B (2006). Microsatellite markers identify three lineages of *Phytophthora ramorum* in US nurseries, yet single lineages in US forest and European nursery populations.. Mol Ecol.

[29] Prospero S, Grünwald NJ, Winton LM, Hansen EM (2009). Migration patterns of the emerging plant pathogen *Phytophthora ramorum* on the west coast of the United States of America.. Phytopathology.

[30] Martin FN (2008). Mitochondrial haplotype determination in *Phytophthora ramorum*.. Curr Genet.

[31] Goss EM, Carbone I, Grünwald NJ (2009). Ancient isolation and independent evolution of the three clonal lineages of the exotic sudden oak death pathogen *Phytophthora ramorum*.. Mol Ecol.

[32] Grünwald NJ, Goss EM, Press CM (2008). *Phytophthora ramorum*: a pathogen with a remarkably wide host-range causing sudden oak death on oaks and ramorum blight on woody ornamentals.. Mol Plant Pathol.

[33] Grünwald NJ, Goss EM, Ivors K, Garbelotto M, Martin FN (2009). Standardizing the nomenclature for clonal lineages of the sudden oak death pathogen, *Phytophthora ramorum*.. Phytopathology.

[34] Grünwald NJ, Goss EM, Larsen MM, Press CM, McDonald VT (2008). First report of the European lineage of *Phytophthora ramorum* on *Viburnum* and *Osmanthus* spp. in a California nursery.. Plant Dis.

[35] Hansen EM, Reeser PW, Sutton W, Winton LM (2003). First report of A1 mating type of *Phytophthora ramorum* in North America.. Plant Dis.

[36] Elliot M, Sumampong G, Varga A, Shamoun SF, James D (2009). PCR-RFLP markers identify three lineages of the North American and European populations of *Phytophthora ramorum*.. For Pathol. In press.

[37] Werres S, Kaminski K (2005). Characterisation of European and North American *Phytophthora ramorum* isolates due to their morphology and mating behaviour in vitro with heterothallic *Phytophthora* species.. Mycol Res.

[38] Ivors KL, Hayden KJ, Bonants PJ, Rizzo DM, Garbelotto M (2004). AFLP and phylogenetic analyses of North American and European populations of *Phytophthora ramorum*.. Mycol Res.

[39] Calinski RB, Harabasz J (1974). A dendrite method for cluster analysis.. Commun Stat.

[40] Bilodeau GJ, Lévesque CA, De Cock AWAM, Brière SC, Hamelin RC (2007). Differentiation of European and North American genotypes of *Phytophthora ramorum* by real-time polymerase chain reaction primer extension.. Can J Plant Pathol.

[41] Tyler BM, Tripathy S, Zhang X, Dehal P, Jiang RH (2006). *Phytophthora* genome sequences uncover evolutionary origins and mechanisms of pathogenesis.. Science.

[42] Goodwin SB (1997). The population genetics of *Phytophthora*.. Phytopathology.

[43] Chamnanpunt J, Shan WX, Tyler BM (2001). High frequency mitotic gene conversion in genetic hybrids of the oomycete Phytophthora sojae.. Proc Natl Acad Sci USA.

[44] Garnica DP, Pinzon AM, Quesada-Ocampo LM, Bernal AJ, Barreto E (2006). Survey and analysis of microsatellites from transcript sequences in *Phytophthora* species: frequency, distribution, and potential as markers for the genus.. BMC Genomics.

[45] Grünwald NJ, Goss EM, Lamour K, Kamoun S (2009). Evolution and Genetics of the Invasive Sudden Oak Death Pathogen *Phytophthora ramorum*.. Oomycete Genetics and Genomics: Biology, Interactions with Plants and Animals, and Toolbox.

[46] Rogers JS (1972). Measures of genetic similarity and genetic distance. Studies in Genetics VII.

[47] Wright S (1978). Evolution and the Genetics of Populations, Vol. 4 Variability Within and Among Natural Populations.

[48] Miller MP (1997). Tools for Population Genetic Analyses (TFPGA) 1.3.. http://www.marksgeneticsoftware.net/.

[49] Tamura K, Dudley J, Nei M, Kumar S (2007). MEGA4: Molecular Evolutionary Genetics Analysis (MEGA) Software Version 4.0.. Mol Biol Evol.

[50] Stoddart JA, Taylor JF (1988). Genotypic diversity: Estimation and prediction in samples.. Genetics.

[51] Grünwald NJ, Goodwin SB, Milgroom MG, Fry WE (2003). Analysis of genotypic diversity data for populations of microorganisms.. Phytopathology.

[52] Ludwig JA, Reynolds JF (1988). Statistical Ecology: A Primer on Methods and Computing.

[53] Szpiech ZA, Jakobsson M, Rosenberg NA (2008). *ADZE*: Allelic Diversity Analyzer Version 1.0.. http://rosenberglab.bioinformatics.med.umich.edu/adze.html.

[54] Excoffier L, Smouse P, Quattro J (1992). Analysis of molecular variance inferred from metric distances among DNA haplotypes: Application to human mitochondrial DNA restriction data.. Genetics.

[55] Excoffier L, Schneider S (2005). Arlequin ver 3.0: an integrated software package for population genetics data analysis.. Evol Bioinform Online.

[56] Bruvo R, Michiels NK, D'Souza TG, Schulenburg H (2004). A simple method for the calculation of microsatellite genotype distances irrespective of ploidy level.. Mol Ecol.

[57] Excoffier L, Smouse PE (1994). Using allele frequencies and geographic subdivision to reconstruct gene trees within a species: molecular variance parsimony.. Genetics.

[58] Gansner ER, North SC (2000). An open graph visualization system and its applications to software engineering.. Software Pract Exper.

[59] Falush D, Stephens M, Pritchard JK (2007). Inference of population structure using multilocus genotype data: dominant markers and null alleles.. Mol Ecol Notes.

[60] Pritchard JK, Stephens M, Donnelly P (2000). Inference of population structure from multilocus genotype data.. Genetics.

[61] Corander J, Marttinen P, Siren J, Tang J (2008). Enhanced Bayesian modelling in BAPS software for learning genetic structures of populations.. BMC Bioinformatics.

[62] Meirmans PG, Van Tienderen PH (2004). genotype and genodive: two programs for the analysis of genetic diversity of asexual organisms.. Mol Ecol Notes.

[63] Beerli P (2004). Effect of unsampled populations on the estimation of population sizes and migration rates between sampled populations.. Mol Ecol.

[64] Beerli P (2007). Estimation of the population scaled mutation rate from microsatellite data.. Genetics.

[65] Beerli P, Felsenstein J (2001). Maximum likelihood estimation of a migration matrix and effective population sizes in *n* subpopulations by using a coalescent approach.. Proc Natl Acad Sci USA.

