# Supplementary material for: Population Genetic Analysis Infers Migration Pathways of Phytophthora ramorum in US Nurseries
Source: PLoS Pathog. 2009 Sep 18;5(9):e1000583. doi: 10.1371/journal.ppat.1000583 (PMC2736564; doi:10.1371/journal.ppat.1000583)
Supplement: Table S1 — Allele sizes at eight loci for each multilocus genotype (MG) observed within clonal lineages EU1, NA1, and NA2. (0.07 MB PDF) [file ppat.1000583.s001.pdf]

Table S1. Allele sizes at eight loci for each multilocus genotype (MG) observed within clonal lineages EU1, NA1, and NA2.

MG	Microsatellite locus							
	PrMS6 $(CGA)_8$	Pr9C3 $(CA)_{15}$	PrMS39b $(GA)_4(GATA)_{33}$	PrMS43a $(CAGA)_{71}$	PrMS43b $(CAGA)_{75}$ (…) $(CAGA)_{17}$	PrMS45 $(TCCG)_{11}$	18 $(AG)_{39}$	64 $(CT)_{16}$
EU1 lineage								
1	165/165	218/224	136/140	162/186	134/145	null	217/261	350/397
2	165/165	218/224	136/140	162/186	134/145	null	217/261	350/401
NA1 lineage								
1	165/168	216/226	246/246	372/372	489/489	166/186	219/275	342/379
2	165/168	216/226	250/250	368/368	485/485	166/186	219/275	342/379
3	165/168	216/226	246/246	372/372	485/485	166/186	219/275	342/379
4	165/168	216/226	250/250	368/368	489/489	166/186	219/275	342/379
5	165/168	216/226	250/250	364/364	485/485	166/186	219/275	342/379
6	165/168	216/226	246/246	368/368	489/489	166/186	219/275	342/379
7	165/168	216/226	246/246	376/376	489/489	166/186	219/275	342/379
8	165/168	216/226	250/250	364/364	485/485	166/186	219/277	342/379
9	165/168	216/226	246/246	372/372	null	166/186	219/275	342/379
10	165/168	216/226	246/246	376/376	485/485	166/186	219/275	342/379
11	165/168	216/226	246/246	372/372	493/493	166/186	219/275	342/379
12	165/168	216/226	254/254	368/368	485/485	166/186	219/275	342/379
13	165/168	216/226	250/250	360/360	485/485	166/186	219/275	342/379
14	165/168	216/226	250/250	368/368	481/481	166/186	219/275	342/379
15	165/168	216/226	250/250	372/372	489/489	166/186	219/275	342/379
16	165/168	216/226	250/250	372/372	485/485	166/186	219/279	342/379
17	165/168	216/226	246/246	372/372	489/489	166/186	219/275	342/383
18	165/168	216/226	250/250	364/364	489/489	166/186	219/275	342/379
19	165/168	216/226	242/242	372/372	485/485	166/186	219/275	342/379
20	165/168	216/226	246/246	368/368	485/485	166/186	219/275	342/379
21	165/168	216/226	246/246	376/376	493/493	166/186	219/275	342/379
22	165/168	216/226	254/254	364/364	485/485	166/186	219/275	342/379
23	165/168	216/226	250/250	349/349	485/485	166/186	219/275	342/379
24	165/168	216/226	250/250	364/364	485/485	166/186	219/279	342/379
25	165/168	216/226	250/250	364/364	489/489	166/186	219/277	342/379
26	165/168	216/226	246/246	376/376	null	166/186	219/275	342/379
27	165/168	216/226	246/246	368/368	493/493	166/186	219/279	342/379
28	165/168	216/226	250/250	360/360	481/481	166/186	219/275	342/379
29	165/168	216/226	246/246	372/372	477/477	166/186	219/275	342/379
30	165/168	216/226	246/246	372/372	481/481	166/186	219/275	342/379
31	165/168	216/226	246/246	372/372	489/489	166/186	219/271	342/379
32	165/168	216/226	250/250	376/376	489/489	166/186	219/279	342/379
33	165/168	216/226	250/250	345/345	481/481	166/186	219/275	342/379

34	165/168	216/226	250/250	364/364	477/477	166/186	219/275	342/379
35	165/168	216/226	246/246	368/368	489/489	166/186	219/275	342/342
36	165/168	216/226	246/246	372/372	418/418	166/186	219/275	342/379
37	165/168	216/226	246/246	372/372	489/489	166/186	219/279	342/379
38	165/168	216/226	250/250	376/376	489/489	166/186	219/275	342/379
39	165/168	216/226	246/246	380/380	485/485	166/186	219/275	342/379
40	165/168	216/226	250/250	372/372	485/485	166/186	219/275	342/379
41	165/168	216/226	246/246	364/364	489/489	166/186	219/275	342/379
42	165/168	216/226	246/246	368/368	406/406	166/186	219/275	342/379
43	165/168	216/226	246/246	368/368	477/477	166/186	219/275	342/379
44	165/168	216/226	250/250	368/368	493/493	166/186	219/275	342/379
45	165/168	216/226	250/250	368/368	null	166/186	219/275	342/379
46	165/168	216/226	250/250	368/368	485/485	166/186	219/277	342/379
47	165/168	216/226	250/250	368/368	489/489	166/186	219/277	342/379
48	165/168	216/226	250/250	368/368	485/485	166/186	219/279	342/379
49	165/168	216/226	250/250	376/376	485/485	166/166	219/279	342/379
50	165/168	216/226	254/254	360/360	485/485	166/186	219/271	342/379
51	165/168	216/226	254/254	364/364	481/481	166/186	219/275	342/379
52	165/168	216/226	254/254	372/372	485/485	166/186	219/275	342/379
53	165/168	216/226	254/254	368/368	485/485	186/186	219/275	342/379
NA2 lineage								
1	165/168	216/216	145/151	157/170	null	null	221/221	344/360
2	165/168	216/216	145/151	161/174	null	null	221/221	344/360
