# Supplementary material for: Population Genetic Analysis Infers Migration Pathways of Phytophthora ramorum in US Nurseries
Source: PLoS Pathog. 2009 Sep 18;5(9):e1000583. doi: 10.1371/journal.ppat.1000583 (PMC2736564; doi:10.1371/journal.ppat.1000583)
Supplement: Table S2 — Distribution of multilocus genotypes belonging to the NA1 clonal lineage. (0.06 MB PDF) [file ppat.1000583.s002.pdf]

Table S2. Distribution of multilocus genotypes belonging to the NA1 clonal lineage.

NA1 multilocus genotype	Num. of isolates	States (number isolates per state)
1	52	AL (1), AR (1), CA (20), FL (5), GA (11), LA (1), MD (1), NM (1), OR (1), PA (1), SC (1), TX (3), VA (2), WA (3)
2	22	CA (1), CT (3), OR (6), WA (12)
3	14	CA (3), NC (2), TX (1), WA (8)
4	12	CA (1), OR (2), WA (9)
5	10	CT (1), OR (4), WA (5)
6	9	FL (1), GA (7), VA (1)
7	8	CA (2), GA (2), OR (1), TN (1), TX (1), WA (1)
8	8	WA
9[a]	7	CA (6), WA (1)
10	6	AL (1), MS (1), WA (4)
11	6	CA (1), CO (1), FL (1), GA (2), TX (1)
12	6	OR (2), WA (4)
13	5	CT (1), OR (2), WA (2)
14	5	WA
15	4	FL (1), GA (1), WA (2)
16	4	WA
17	3	GA (1), WA (2)
18	3	OR (2), WA (1)
19	3	WA
20	3	WA
21	2	GA
22	2	GA (1), OR (1)
23	2	OR
24	2	OR (1), WA (1)
25	2	WA
26[a]	1	CA
27	1	CA
28	1	CT
29	1	FL
30	1	FL
31	1	GA
32	1	GA
33	1	OR
34	1	OR
35	1	SC
36	1	TX
37	1	TX
38	1	TX
39	1	VA
40	1	VA
41	1	WA

42	1	WA
43	1	WA
44	1	WA
45[a]	1	WA
46	1	WA
47	1	WA
48	1	WA
49	1	WA
50	1	WA
51	1	WA
52	1	WA
53	1	WA

[a] Genotype with null allele at PrMS43b
