# Supplementary material for: Population Genetic Analysis Infers Migration Pathways of Phytophthora ramorum in US Nurseries
Source: PLoS Pathog. 2009 Sep 18;5(9):e1000583. doi: 10.1371/journal.ppat.1000583 (PMC2736564; doi:10.1371/journal.ppat.1000583)
Supplement: Table S3 — Confirmed P. ramorum-positive nursery-related sites (nurseries and residential landscapes, except for CA which is nurseries only) by state and year (http://www.suddenoakdeath.org). (0.06 MB PDF) [file ppat.1000583.s003.pdf]

Table S3. Confirmed *P. ramorum*-positive nursery-related sites (nurseries and residential landscapes, except for CA which is nurseries only) by state and year (http://www.suddenoakdeath.org).

State	Year	*Pr*+ Sites
California (CA)	2004	55
	2006	28
	2007	7
Colorado (CO)	2004	1
Connecticut (CT)	2004	3
	2006	1
Florida (FL)	2004	6
	2006	2
	2007	1
Georgia (GA)	2004	16
Louisiana (LA)	2004	5
Maryland (MD)	2004	3
Mississippi (MS)	2007	1
N. Carolina (NC)	2004	9
New Mexico (NM)	2004	1
Oregon (OR)	2004	24
	2005	20
	2006	13
	2007	2
Pennsylvania (PA)	2004	2
S. Carolina (SC)	2004	4
Tennessee (TN)	2004	2
Texas (TX)	2004	11
Virginia (VA)	2004	2
Washington (WA)	2004	25
	2005	16
	2006	12
	2007	7
