# Supplementary figures and images for: Population Genetic Analysis Infers Migration Pathways of Phytophthora ramorum in US Nurseries
Source: PLoS Pathog. 2009 Sep 18;5(9):e1000583. doi: 10.1371/journal.ppat.1000583 (PMC2736564; doi:10.1371/journal.ppat.1000583)

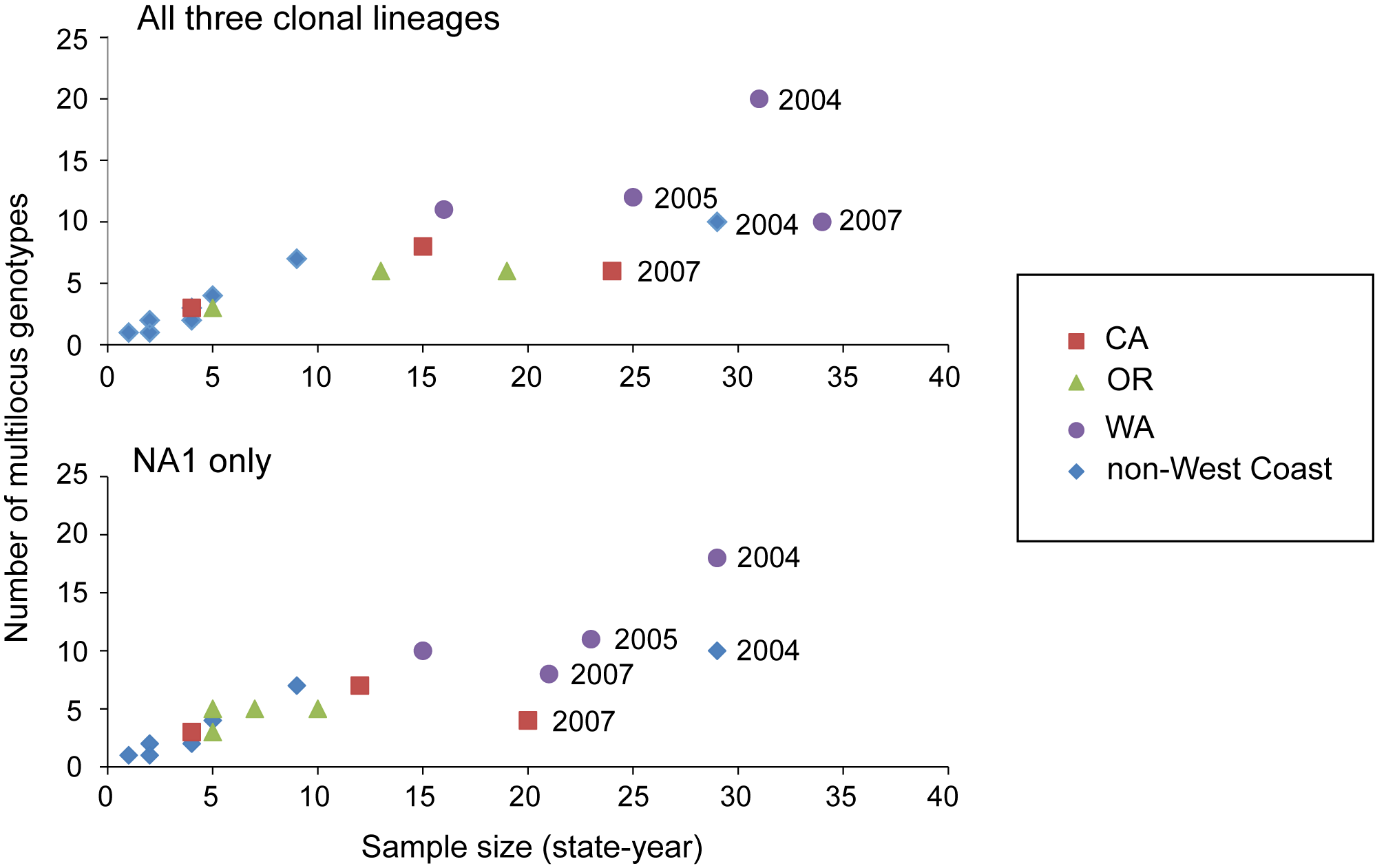

Supplement: Figure S1 — Relationship between sample size and number of multilocus genotypes in the sample. Samples are isolates from one state in one year. The upper graph shows this relationship when multilocus genotypes from all three clonal lineages are considered. The lower graph shows the number of multilocus genotypes in the NA1 lineage only. Sample year is shown for the largest sample sizes to the right of the marker. (0.29 MB TIF) [file ppat.1000583.s004.tif]
